# Pathogen invasion and non-epizootic dynamics in Pacific newts in California over the last century

**DOI:** 10.1371/journal.pone.0197710

**Published:** 2018-07-02

**Authors:** Shruti Chaukulkar, Hasan Sulaeman, Andrew G. Zink, Vance T. Vredenburg

**Affiliations:** Department of Biology, San Francisco State University, San Francisco, California, United States of America; University of South Dakota, UNITED STATES

## Abstract

Emerging infectious disease is a growing threat to global biodiversity. The infectious disease chytridiomycosis, caused by the fungal pathogen *Batrachochytrium dendrobatidis (Bd)* has led to the decline and extinction of hundreds of amphibian species. Severe *Bd-*caused epizootics have been documented in North, Central and South America—with many of the research focused on anurans. California, where *Bd*-related epizootics and amphibian declines have been reported, has some of the highest diversity of salamanders. After more than a decade since the first known epizootic in California, little is known about *Bd* disease dynamics in salamanders. Pacific newts (Genus: *Taricha*) are ideal study species because of their abundance, wide geographic range, occurrence in both aquatic and terrestrial habitats, and how little is known about *Bd* infection dynamics for this group. We conducted a retrospective study to determine the relationship between Pacific newts and the fungal pathogen. We tested 1895 specimens collected between 1889–2009 and found no evidence of *Bd-*infected Pacific newts until the late 1940’s. Although we estimate that *Bd* emerged in this genus and rapidly spread geographically throughout California, we did not find evidence for epizootic dynamics. *Bd* infection prevalence and intensity, two measures commonly used to estimate dynamics, remained consistently low over time; suggesting Pacific newts may not be highly susceptible. Also, we found the timing of first *Bd* emergence in Pacific newts predate *Bd* emergence in other California salamander species. In addition, we found several environmental and anthropogenic factors correlated with *Bd* prevalence which may help explain *Bd* disease dynamics in the genus *Taricha*. Pacific newts may be a reservoir species that signal pathogen invasion into California salamanders, though further studies are needed.

## Introduction

Amphibian population declines have occurred globally beginning in the late 1970’s [1,2]. While there are many causes for the declines, emerging infectious disease is one of the main factors [3]. This phenomenon is considered the worst case of disease caused die-offs recorded and is attributable to a single pathogen [4]. The fungal pathogen, *Batrachochytrium dendrobatidis*, was first discovered in in dead and dying frogs in Central America [5, 6], and later in North America, Europe and Australia. [7, 8, 4, 9], and causes the disease chytridiomycosis. *Bd* infects the skin of the amphibians and causes hyperkeratosis. Hyperkeratosis is the thickening of the amphibian skin that disrupts the osmotic balance as the infection moves across the skin, leading to death by cardiac arrest [6, 10–12]. In the palmate newt (*Lissotriton helveticus*), *Bd* was shown to decrease ventral water absorption rates after repeated exposure to *Bd* [13].

Emergence and dynamics of *Bd*, since its discovery, are still not fully understood. For example, it has been debated whether *Bd* is an invasive pathogen [14, 15, 8, 9], or whether it was already present globally and only recently became pathogenic [16]. Other factors that influence disease dynamics, such as transmission, host vulnerability, and pathogen strain variation, are also not fully understood. Several studies have shown the rapid invasion of *Bd* through Mexico and Central America [17,8] resulting in epizootics that caused many extinctions [18]. Unfortunately, many declines occurred before *Bd* was described [17]. Retrospective studies analyzing the presence of the pathogen on museum-collected specimens can help create a timeline for disease emergence and transmission. Previous historical studies in California found *Bd* infected amphibians long before the pathogen was first described. Huss et al., 2014 found *Bd*-positive *Rana catesbeiana* in 1928, but found no evidence that epizootics immediately followed. Other studies found *Bd* first appearing in specimens collected in California in the late 1950s, 1960s, and 1970s [19, 20, 21]. The emergence patterns of *Bd* in both frogs and salamander specimens led to the conclusion that *Bd* emerged as a novel pathogen in California [19, 20, 21] and help explain epizootics documented in other species [9]. These retrospective data improve our understanding of *Bd* dynamics and with further study may help identify the origin of *Bd*.

We propose that Pacific newts may be reservoir species for *Bd*, similar to Pacific chorus frogs (*Hyliola regilla*) [22]. A reservoir species maintains the pathogen in hosts and can spread the pathogen to other host species [22]. Pacific newts are abundant and widespread in California, migrate and have a large home range. There are currently four known species of pacific newts; *Taricha torosa* (California newt), *Taricha granulosa* (Rough skin newt), *Taricha rivularis* (Red-bellied newt) and *Taricha sierrae* (Sierra newt) [23]. Pacific newts also utilize both aquatic and terrestrial habitats and have a short larval stage of 4–6 months in water followed by metamorphosis and a move to terrestrial habitats until they reach sexual maturity. [24,25]. Pacific newts are completely aquatic during breeding season and move to a terrestrial habitat until the next breeding cycle [26, 27, 28]. Additionally, there are over 9,000 specimens collected from 1880–2015 and are currently housed in permanent museum collections. This allows for random sampling of museum specimens to provide a better insight on *Bd* emergence. We conducted a randomly sampled retrospective survey using the museum specimens to describe the spread of *Bd* across California on the genus *Taricha*. We also evaluated historical *Bd* prevalence and intensity in association with several biotic and abiotic factors that would affect the ecology of *Bd*. Lastly, we used a Bayesian analysis to estimate the time of invasion [21, 29]. From this study, we discovered various insights into the temporal and spatial dynamics of *Bd* on Pacific newts in California, along with various ecological drivers of *Bd* infections.

## Materials and methods

### Museum sampling

In order to create the timeline for *Bd* prevalence, we created a sampling regime from the specimen database for Taricha (n = 9,774) from VertNet.org. The samples were selected from the permanent collections housed at the Museum of Vertebrate Zoology, University of California, Berkeley and the California Academy of Sciences, San Francisco. Samples were randomly selected in a blocked design, where 20 replicate samples per species, per decade (1889–2009) were selected for skin swab collection. The selection process led to total of 1895 museum specimens. These specimens were then sampled for *Bd* presence. The swabbing and qPCR results from our experiment can be accessed on the amphibian disease portal [30].

### Swab processing

To reduce cross-contamination between specimens kept in the same jar at the natural history museums, every specimen was rinsed with 70% EtOH prior to swabbing and then swabbed 30 times using a sterile medical swab (MW113, Medical Wire and Equipment, Corsham, UK) across its dorsal and ventral surfaces along with the toes and the mouthparts; changing gloves between each specimen. Swabs collected were kept dry in 1.5 mL microcentrifuge tubes at 4°C until processed. Before extraction process, swabs were dried in a Spin Vac (Savant Instruments, Farmingdale, NY, USA) to remove EtOH. DNA Extraction was performed using 40μL of Prepman Ultra (Applied Biosystems, Carlsbad, CA, USA) [17, 31, 32] and diluted 1:10 with 0.25 × TE buffer. We analyzed each sample in singlicate, using 5 μL of the diluted DNA extract. When run in singlicate, on specimens identified as Bd-infected from histological examination, qPCR correctly detected *Bd* 60% of the time [17]. Universal DNA standards from the global pandemic lineage strain (provided by A.S. Hyatt) were used to calibrate the qPCR (0.1, 1.0, 10, and 100 zoospore equivalents per reaction). Negative controls were also included during extraction and qPCR to detect contamination. Samples were run on an Applied Biosystems 7300 Real-Time PCR thermocycler. We calculated the number of zoospores in terms of Zswab (i.e., estimated *Bd* zoospore genomic equivalents on each swab) by multiplying qPCR results by 80 to account for sample dilution (40 μL Prepman × 10 dilution/ 5 μL for reaction = 80). A *Bd*-positive sample was described as having a Zswab score greater than zero.

### Statistics

To characterize the temporal and spatial distribution of *Bd* on Pacific newts in California, we calculated 95% confidence intervals for *Bd* prevalence for the genus *Taricha* we sampled from each decade based on a binomial probability distribution. To estimate the arrival date of *Bd* in the California, we used a Bayesian modeling approach. In this model, the process of *Bd* arrival is described using a threshold model where *Bd* switches from absent to present with some mean prevalence. The number of infected newts in each year was treated as a draw from a binomial distribution with a sample size equal to the number of newts sampled in that year [29, 21]. We also calculated the probability of detecting zero positives for each decade prior to the decade of first detection to check if *Bd* was present prior to first detected positive in California. These calculations are based on the binomial distribution and utilizes the total number of samples evaluated in a particular decade as the number of trials. A previous study that used a qPCR technique to depict *Bd* endemism in North American museum specimens found that 11% of specimens tested positive for *Bd* [33] Therefore, we used 0.11 as the “true” probability of finding a *Bd-*positive individual for our binomial confidence interval.

All statistical analyses were performed using the statistical software R (version 3.4.2). We did a linear regression for *Bd* infection status as a response variable, assuming a binomial distribution as animals can only either be infected or not infected. We used the following explanatory variable groups for our response variable: human footprint, precipitation, temperature, distance to water body, elevation, amphibian species richness, and soil-water balance. Elevation and topographic information were extracted from USGS (nationalmap.gov, https://nhd.usgs.gov/data.html), soil-water balance data was used from the consortium for spatial information (http://www.cgiar-csi.org/data), temperature and precipitation data was used from WorldClim (http://www.worldclim.org). We include human footprint data from a recent study that creates a human footprint index based on anthropogenic factors (e.g. human population size, light pollution, number of roads and railways, etc.) [34]. To reduce the number of factors, we first did a Pearson correlation test to eliminate highly correlated factors (r > 0.9 or < -0.9). We then performed a stepwise regression to choose the best-fit model based on the AIC [35, 36].

## Results

### Museum sampling

**Fig 1:**
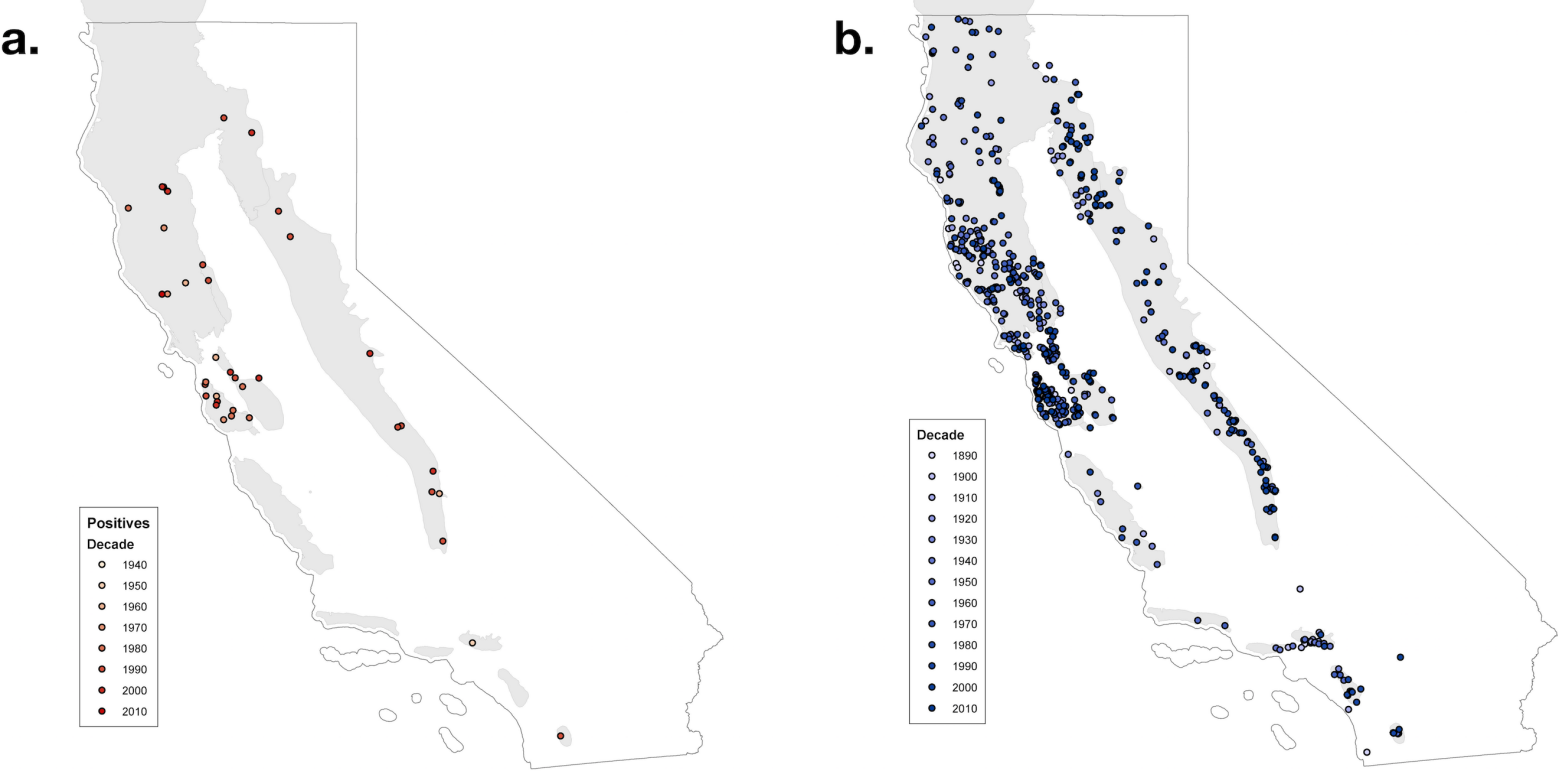
(a) Spatial and temporal distribution of Pacific newts in California that tested positive for *Bd* from 1889–2009. (b) Spatial and Temporal distribution of Pacific newts in California that tested negative for *Bd* from 1889–2009.

**Fig 2:**
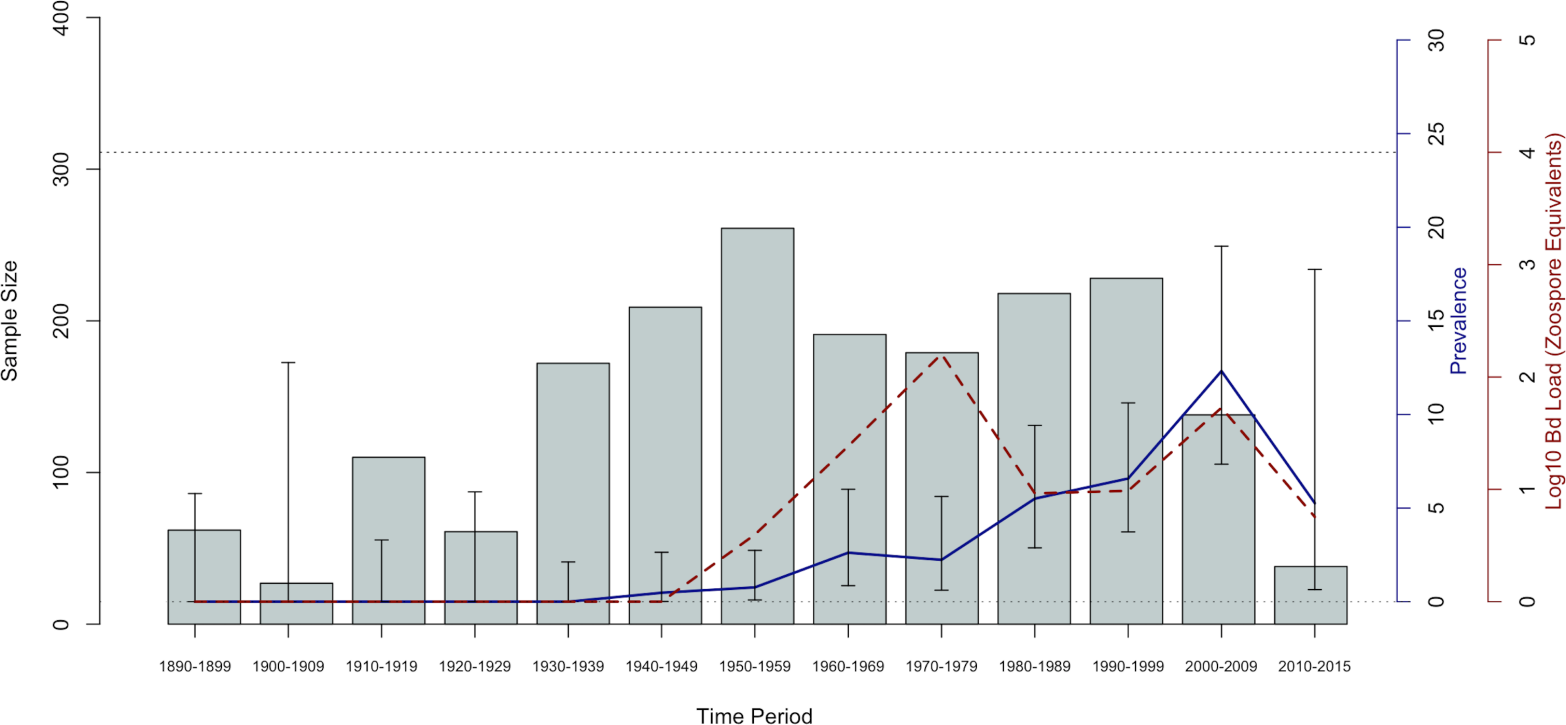
Emergence of *Bd* in Pacific newts from 1889–2009. Infection prevalence (solid line) and infection intensity (broken line) patterns over time. Gray bars represent number of samples analyzed per decade. Infection intensity is represented with a broken line, while the dashed line at Log_10_ Zscore = 4 represents the 10,000 zoospore genomic equivalents shown to be associated with mortality in anurans as a basis for comparison.

Of the 1895 archived Pacific newt specimens in the retrospective study, 58 tested positive for *Bd*, with an overall prevalence of 3.06% (Figs 1 and 2). The earliest positive was a 1948 specimen in San Diego County and since then, *Bd* has spread throughout California over time (Fig 1). The Bayesian analysis gave 95% credible interval for the date of *Bd* arrival in Californian Pacific newts between 1945 to 1948 with a post-arrival *Bd* prevalence between 4% to 5% (mean: ~4.5%). Death follows in adult frogs when the individual reaches an infection intensity of 10,000 zoospores. Though there are currently no data showing a similar threshold for salamanders, we listed 10,000 zoospores as a measure of comparison for a potentially deadly infection load on an individual [9, 37, 17]. None of the infected individuals from the museum study had infection intensities greater than the 10,000 zoospore genomic equivalents value associated with mortality in anurans. Based on the binomial distribution calculation, the probability of finding no *Bd*-positive samples in each decade was less than 0.001 (Table 1).

**Table 1:** *Batrachochytrium* dendrobatidis (*Bd*) prevalence in all four species of Pacific newts.

Decade	Positive Individuals	Sample Size	% Positive	Lower CI	Upper CI	Pr (noBd)*
1890	0	62	0.00	0	0.06	< 0.001
1900	0	27	0.00	0	0.13	0.043
1910	0	110	0.00	0	0.03	< 0.001
1920	0	61	0.00	0	0.06	< 0.001
1930	0	172	0.00	0	0.02	< 0.001
1940	1	209	0.48	< 0.01	0.03	< 0.001
1950	2	261	0.77	< 0.01	0.03	< 0.001
1960	5	191	2.62	< 0.01	0.06	< 0.001
1970	4	179	2.23	< 0.01	0.06	< 0.001
1980	12	218	5.50	0.03	0.09	< 0.001
1990	15	228	6.58	0.04	0.11	< 0.001
2000	17	138	12.32	0.07	0.19	< 0.001
2010	2	38	5.26	< 0.01	0.18	0.011
Total	58	1894	3.06			

Museum specimens collected in California. Pr(no *Bd*) is the probability of finding no *Bd*-positive samples in each decade (based on a binomial distribution) if *Bd* was actually present with a prevalence of 11% (as seen in enzootic state for over a century in Talley et al. 2015).

*Based on a binomial distribution with a “true” probability of 0.11 (95% confidence interval)

### Linear regression

In the model with the best AIC (AIC = -6680.91), we found that infection status has a positive relationship with the following factors: elevation and annual precipitation (p<0.01, p = 0.03; respectively). We found that infection status has a negative relationship with the following factors: precipitation of the driest quarter and mean actual evapotranspiration (p = 0.01, p = 0.01; respectively). Infection status was not shown to have a significant relationship with the following factors: snout to vent length, maximum temperature of the warmest month (Table 2).

**Table 2:** Linear regression output. Environmental factors and their relationship to *Bd* presence.

Variables	Model 1	Model 2	Model 3	Model 4	Model 5
Snout to Vent Length	X	X	X	X	X
Elevation (+)	X	X	X	X	X
Mean precipitation (+)	X	X	X	X	X
Precipitation of driest quarter (-)	X	X	X	X	X
Mean actual evapotranspiration (-)	X	X	X	X	X
Max temperature of warmest month	X				
Min temperature of coldest month				X	X
Annual mean temperature			X	X	X
Railways					X
AIC	-6680.91	-6680.87	-6680.55	-6679.47	-6678.21
Δ AIC	0.04	0.33	1.07	1.26	NA

(+) indicate a positive relationship between factor and *Bd* presence

(-) indicate a negative relationship between factor and *Bd* presence

The model with the lowest AIC value (1^st^ model) was considered the best fit model.

## Discussion

*Bd* has been associated with various amphibian declines in multiple regions throughout the world [8, 9]. Museum samples offer evidence of the timing as well as location of the pathogen arrival leading to amphibian decline [17]. However, museum specimens were collected for reasons unrelated to our study, and thus the specimen collections contain sampling biases that are not related to our study. The emergence of *Bd* in the 1960's in our samples and its following rise in prevalence concurs with other known die offs in California beginning in the 1970's [38, 39, 9, 20, 21]. Our earliest positive was from 1948, though it is possible that there are earlier positives we didn’t detect from a different *Bd* strain [40]. In addition, it is also possible that *Bd* was already present but at such low prevalence that no die offs were recorded and most species were not found to be infected. However, with our robust sample size up to our first Bd infected animal (n = 641 before 1950), we have sufficient power to detect even a very low prevalence (Table 1). Our Bayesian analysis predict the arrival of the *Bd* strain we tested for between 1944–1948 in California Pacific newts with a mean prevalence of around 4%. This supports our hypothesis that Pacific newts may be a reservoir species for chytridiomycosis (at least in terrestrial salamanders) [22]. As a Bd reservoir, newts would help maintain and spread Bd to other hosts [41]. Specimens collected prior to 1940 (432 samples) tested negative, consistent with the hypothesis that *Bd* emerged as an epizootic in California. Our study suggests that *Bd* possibly spread throughout California with multiple points of entry considering the distance between the earliest positive to present day (Fig 1A and 1B). This coincides with the wide spread geographic range and use of multiple habitats of Pacific newts. Recently, a new chytrid fungus specific to salamanders, *Batrachochytrium salamandrivorans* (*Bsal*) was described in the Netherlands during a mass die-off in the European fire salamanders. [42]. *Bsal* poses a major threat to the salamander diversity in North America [42, 43] and has not been shown to be present in North America. Therefore, there is a need for further studies regarding Pacific newts’ possible role as a reservoir species for the pathogen.

Consistent with other studies, our linear regression output found that infection status had a positive relationship with elevation [44–46]. It is interesting given that we had a relatively small range of elevations (mean = 443 meters; range = 2-1800m). The upper elevation limit in our sampling is due to Pacific newts’ elevation limit of 2000m [47]. Our results also suggest a negative relationship between *Bd* infection and precipitation of the driest quarter and a positive relationship between *Bd* infection and annual precipitation, consistent with past studies [16, 44, 48, 49]. Lastly, we found that mean actual evapotranspiration, the rate of which the water in the soil evaporates, had a negative relationship with *Bd* infection. *Bd* has been known to reside and even to survive in moist soils [50, 51], thus soil run-offs and soil transport (e.g. during construction or landscaping being described as possible means to spread *Bd* [52, 53]. Consequently, higher soil evapotranspiration translates to a drier soil and a less suitable environment for *Bd*. Our linear regression analysis is limited by data availability for some of the variables (e.g. human footprint).

Our study focuses on four newt species that occur along western North America, where *Bd* epizootics have been documented [9]. However, there are several field surveys of *Bd* infections in newts from other regions where *Bd* epizootics are not known. For example, a study in the Eastern newt complex (*N*. *viridescens*) showed *Bd*-infections in wild populations had low zoospore counts and low prevalence [54, 55, 56, 57]. Another study suggested that Eastern newts may act as a *Bd* reservoir [56] and can develop acquired *Bd*-immunity as they mature [57]. These studies in other systems provide a framework for understanding how Bd may interact with other salamanders in the family Salamandridae, but comparisons must be done with the understanding that those species may have different evolutionary relationships with this pathogen. For example, there are no known epizootics of *Bd* where Eastern newts occur, and some have suggested that *Bd* may have a longer evolutionary history with amphibian species on Eastern US [33].

In our study, we provide new evidence that *Bd* is a novel pathogen in California, suggesting that *Bd* emerged in the last 4–5 decades. This is important because the pathogen was described almost 20 years after the first mass die offs were reported in California [21, 38]. In this study we found evidence that Bd invaded and became established in populations of Pacific newts earlier than other salamander species in the California region. Pacific newts have a widespread geographic range, use multiple habitats (aquatic/terrestrial) for extended periods of time (i.e. months), have large home range size, and have large populations. We found the pattern of emergence, where *Bd* was absent and then spread geographically (Fig 1) and increased in prevalence over time, to be similar to that found in other salamanders in the region. However, we also found that *Bd* dynamics in Pacific newts seem to represent a non-epizootic dynamic, where *Bd* infection intensities remain low. This may indicate that Pacific newts may not experience epizootic conditions in nature. We suggest that additional studies including laboratory and field-based *Bd* susceptibility studies are necessary to fully describe the relationship between *Bd* and Pacific newts and whether or not Pacific newts would make for a reservoir species that maintains the pathogen in amphibian communities.
